# ppiGReMLIN: a graph mining based detection of conserved structural arrangements in protein-protein interfaces

**DOI:** 10.1186/s12859-020-3474-1

**Published:** 2020-04-15

**Authors:** Felippe C. Queiroz, Adriana M. P. Vargas, Maria G. A. Oliveira, Giovanni V. Comarela, Sabrina A. Silveira

**Affiliations:** 1 Department of Computer Science, Universidade Federal de Viçosa, Av Peter Henry Rolfs, Viçosa, MG, Brazil; 2 Department of Biochemistry and Molecular Biology, Universidade Federal de Viçosa, Av Peter Henry Rolfs, Viçosa, MG, Brazil; 3 Instituto de Biotecnologia aplicada a Agropecuaria, BIOAGRO-UFV, Av Peter Henry Rolfs, Viçosa MG, Brazil; 4 Department of Computer Science, Universidade Federal do Espírito Santo, Av Fernando Ferrari, Vitória, ES, Brazil; 5 European Molecular Biology Laboratory, European Bioinformatics Institute (EMBL-EBI), Hinxton, CB10 1SD UK

**Keywords:** Protein-protein interaction, Data mining, Interaction patterns

## Abstract

**Background:**

Protein-protein interactions (PPIs) are fundamental in many biological processes and understanding these interactions is key for a myriad of applications including drug development, peptide design and identification of drug targets. The biological data deluge demands efficient and scalable methods to characterize and understand protein-protein interfaces. In this paper, we present ppiGReMLIN, a graph based strategy to infer interaction patterns in a set of protein-protein complexes. Our method combines an unsupervised learning strategy with frequent subgraph mining in order to detect conserved structural arrangements (patterns) based on the physicochemical properties of atoms on protein interfaces. To assess the ability of ppiGReMLIN to point out relevant conserved substructures on protein-protein interfaces, we compared our results to experimentally determined patterns that are key for protein-protein interactions in 2 datasets of complexes, Serine-protease and BCL-2.

**Results:**

ppiGReMLIN was able to detect, in an automatic fashion, conserved structural arrangements that represent highly conserved interactions at the specificity binding pocket of trypsin and trypsin-like proteins from Serine-protease dataset. Also, for the BCL-2 dataset, our method pointed out conserved arrangements that include critical residue interactions within the conserved motif LXXXXD, pivotal to the binding specificity of BH3 domains of pro-apoptotic BCL-2 proteins towards apoptotic suppressors. Quantitatively, ppiGReMLIN was able to find all of the most relevant residues described in literature for our datasets, showing *precision* of at least 69% up to 100% and *recall* of 100%.

**Conclusions:**

ppiGReMLIN was able to find highly conserved structures on the interfaces of protein-protein complexes, with minimum support value of 60%, in datasets of similar proteins. We showed that the patterns automatically detected on protein interfaces by our method are in agreement with interaction patterns described in the literature.

## Background

Protein-protein interactions (PPI) are fundamental in many biological processes, including metabolism, information processing, decision making, transport, and structural organization. They are essential on the understanding of cellular physiology and constitute intricate networks of interactions which produce highly organized, dynamic cellular systems [1, 2].

There are a variety of online resources, such as databases and functional genomics networks, devoted to protein interactions. Some databases, such as HINT [3], BioGRID [4] and APID [5], build genomic networks that cover protein interactions experimentally determined. Other resources, such as ConsensusPathDB [6], IMP [7], IID [8], STRING [9], FunCoup [10] and GeneMANIA [11], include not only experimentally determined protein interactions but also predicted ones (coupled with quality or confidence scores) in an attempt to increase coverage.

These resources support the process of knowledge discovery on protein interactions. In this context, Li and colleagues devised, in [12], a human protein-protein interaction network that enabled to interpret approximately 4,700 genomes and genes involved in autism. The interactions between proteins were considered at the molecular level and used to study the human interactome. This kind of study is relevant because despite being feasible to experimentally test large sets of proteins for thousands of interactions, only relatively small portions of the interactome of many organisms is elucidated by recent screens for protein-protein interactions (for instance, 4-22% of the human interactome [13, 14]). Thus, the computational integration of heterogeneous databases, involving different organisms and different interaction experiments produce protein networks that help to increase coverage of interactomes and improve the quality of annotations.

Another interesting example of knowledge discovery on protein interactions is the focus on understanding the potential of PPIs as targets, which has been addressed by many studies as [15–19]. Here we mention a few interesting findings on this topic. Protein–protein interactions are involved in the regulation of biological systems and can thus be implicated in a number of diseases, which makes PPIs potential targets of great therapeutic interest. PPIs are attractive yet challenging pharmaceutical targets. The identification of inhibitors for protein interactions is considered challenging due to the shallow and extended nature of PPI interfaces [20]. Also, in the interaction between two peptides there is not a well defined binding site and the peptides involved can be intrinsically disordered when not in complex [19]. Compared to classical protein-ligand interactions (PLI), that target one large pocket, PPI inhibitors are smaller in volume and tend to target several pockets [21]. The development of PPI inhibitors aims to mimic peptides, using mimetics that are peptide or non-peptide based [22].

In this work, we are particularly interested in protein-protein interactions in a fine degree of granularity, more specifically, in the residue and even atom level. In this context, Khashan and colleagues [23] proposed SPIDER, a scoring function for docking experiments based on frequent interaction patterns between residues in protein-protein interfaces. It uses a graph representation of interactions based on contact information at the residue level along with Almost-Delaunay tessellation to produce a set of graphs representing the interface. Finally, graph mining techniques were employed over the set of graphs in order to detect frequent patterns of interacting residues.

Morozova and colleagues [24], in contrast, focused on patterns between protein-RNA interactions. In this case, structural information from protein-RNA complexes was used to unveil patterns in nucleoside binding pockets. A three-dimensional superimposition strategy was devised based on the interatomic non-covalent interactions between each RNA base and protein structures obtained from PDB. The computation of interactions was performed considering pyshicochemical properties of the atoms and a distance cutoff. The method was able to single out frequent interactions in nucleoside structure that are discriminant to the binding of each base.

Melo and colleagues [25], in turn, proposed a method based on contact map matching to uncover relevant patterns in protein-protein interactions. The algorithmic approach was based on inter-chain contact maps of protein residues and used image processing to detect conserved interactions in protein-protein interfaces. The authors were able to identify important contacts in complexes for a set of protein families.

A variety of methods were devised to characterize, understand and detect patterns on protein-protein interfaces. Nonetheless, in spite of the relevant contributions of the majority of the works, strategies that depend on multiple structural superimposition might be prohibitively expensive for large scale processing. As the amount of biological data has been growing in a fast pace, scalable techniques are pivotal to perform such tasks in a real-world scenario. Furthermore, strategies that work at the residue granularity might not reveal the specific details involved in the molecular recognition process that takes place on protein-protein interfaces.

To overcome these challenges, this article proposes ppiGReMLIN, a graph based strategy to detect conserved structural arrangements on protein-protein interactions. By structural arrangements, we consider not only specific interacting residues in PPIs, but more general biding motifs that may disclose more information on how interactions occur. Protein-protein interfaces are modeled as graphs in which atoms and non-covalent interactions represent nodes and edges respectively. Nodes and edges are labeled according to their physicochemical properties and distance criteria. The resulting graph dataset is encoded as feature vectors, which serve as input for a clustering analysis. The resulting set of clusters go through a frequent subgraph mining (FSM) to reveal substructures that are conserved across the whole dataset of protein-protein interactions. ppiGReMLIN requires a set of protein-protein complexes involving proteins of interests and is derived from GReMLIN (GRaph Mining strategy to infer proten-Ligand INteraction patterns) [26], which is a graph-based strategy successfully used to infer patterns in protein-ligand interactions. Our method does not rely on sequence alignment nor structural superimposition, and can be used in large-scale datasets of protein interactions.

ppiGReMLIN is based on ideas developed in some of our previous works, concerning the defense of plants against insects and pathogens. The insect *Anticarsia gemmatalis Hübner* is a pest that attacks soybean. The plant, when injured by this insect, produces the Kunitz trypsin inhibitor (KTI), which impairs the process of proteases degradation in the caterpillar gut [27, 28]. Inspired by this natural inhibitor produced by soybean, we are interested in proposing peptides or mimetic peptides to inhibit the proteases of the insect gut, that is, to carry out the ecological control of this pest insect. We used to perform the peptide design manually, with the support of some bioinformatics tools. ppiGReMLIN aims to provide the conserved substructures that should be used in the peptide design process, so that it is performed in a guided way, resulting in peptides or mimetic peptides that can potentially inhibit proteases of the *Anticarsia* gut. It is important to note that our method is not specific to soybean and its insect pest *Anticarsia gemmatalis Hübner* and can be used in other datasets involving protein-protein complexes.

## Methods

**Fig. 1:**
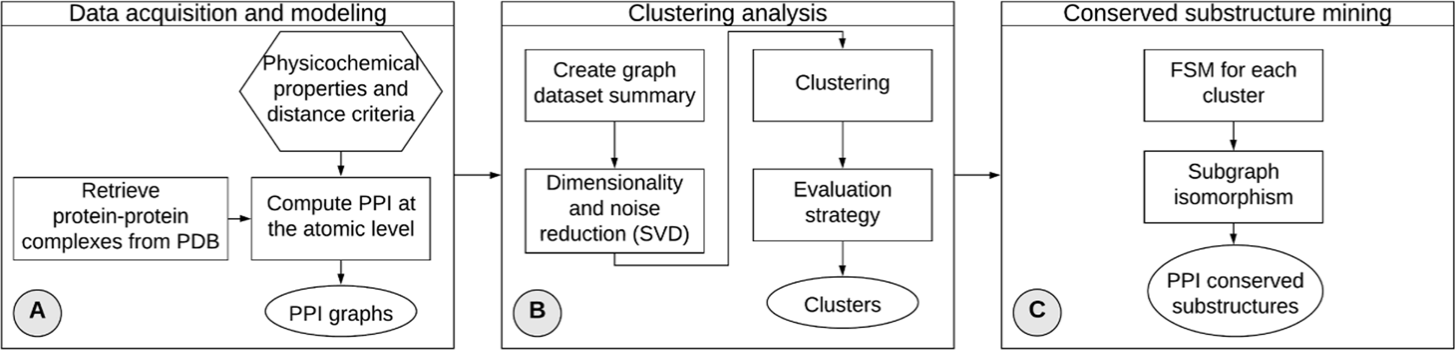
ppiGReMLIN workflow. The workflow is composed of three blocks: Data acquisition and modeling; Clustering analysis; and Conserved substructure mining. Rectangles indicate processing steps; ellipsoids denote output files; and hexagons represent input files or parameters

This section details ppiGReMLIN, our method to infer conserved structural arrangements on protein-protein interface at the atomic level. We describe the datasets, the problem modeling, the data mining tasks, and the evaluation strategy. Figure 1 presents the workflow that outlines our method.

Interactions on the protein-protein interface are computed for a set of complexes at atomic level according to physicochemical properties of atoms and distance criteria in the *Data acquisition and modeling* block (Fig. 1a). The *Clustering analysis* block (Fig. 1b) takes as input a set of graphs that represent the protein-protein interfaces and segments them in groups containing similar graphs to mine frequent substructures in the next block. The resulting clusters serve as input for the *Conserved substructure mining* block (Fig. 1c), where a FSM task is performed to search for conserved substructures in each group.

### Datasets

We instantiated our strategy with two main datasets of protein-protein complexes obtained from PDB. *Serine protease dataset* is composed of trypsin and trypsin-like proteins coupled with some peptide inhibitor and the *BCL-2 dataset* is composed of protein complexes belonging to the BCL-2 family.

#### Serine protease dataset

*Anticarsia Gemmatalis* (AG) is one of many defoliating caterpillars affecting crop yields in agriculture. In north and south America, it infests mainly soybean, causing damage to plants, and potentially destroying entire crops [29]. Agrochemicals are the most sought after solution to control the population of these caterpillars; however, its use in agriculture is highly debated nowadays due to various issues concerning its toxicity not only to the environment, but also to human health. In addition, the employment of these products may cause the surge of other resistant populations of new parasites, which requires the use of even stronger products or higher dosages. One alternative method to circumvent the aforementioned issues consists in the inhibition of the digestive proteases in the caterpillar, which was shown to reduce its growth rate and survival ratio [30, 31].

The basis to construct the Serine protease (SP) dataset was a protease sequence from caterpillar digestive system sequenced in our previous work [32], available at GenBank [33]. We searched the Protein Data Bank (PDB) [34] for proteins with at least 30% sequence similarity using our protease sequence as query. Then, these PDBs were filtered resulting in structures containing a protease chain complexed with some other protein or peptide. This process resulted in 93 PDB entries, which contain mainly serine proteases in complex with some peptide inhibitor. The similarity between inhibitors was not taken into account because we are interested in detecting protein-protein patterns that characterize the caterpillar protease inhibition process.

#### BCL-2 dataset

The BCL-2 proteins are key regulators of programmed cell death, acting in the regulation of cytochrome *c* release on the mitochondrias [35, 36]. They consist in a set of proteins that share homology with the BCL-2 (BH) domains (1-4), being divided into two main sub-groups: the anti-apoptotic proteins and the pro-apoptotic proteins. The former ones are composed by BH domains 1-4, which act preserving the outer mitochondrial membrane integrity by inhibiting its pro-apoptotic counterparts. The pro-apoptotic ones, on the other hand, may have multiple BH domains (such as BAK, BAX, BOK) or just a single BH3 domain (such as BIM, PUMA, NOXA, BID, etc.) Ultimately, the cell status depends on the balance of the levels of these proteins, whose expression can be modulated by means of cell signaling to tip the equilibrium towards survival or death [37]. Indeed, apoptosis has been established as a critical tumor suppression mechanism [38]. Also, a cell’s ability to evade apoptosis alongside with oncogenic mutations that deregulate cell growth and cell cycling greatly enhance tumorigenesis, therefore characterizing evasion from apoptosis as one of the ‘Halmarks of Cancer’ [39, 40].

For this dataset, we queried PDB for proteins with a minimum of 30% sequence similarity with the structure of Mcl-1 (extracted from pdb id 2KBW), an anti-apoptotic human protein of the BCL-2 family. Results were then filtered down to structures containing the query sequence target protein complexed with some other protein. In this proccess, 72 PDB entries were found, which composes our BCL-2 dataset.

### Modeling PPIs as graphs

We computed protein-protein interactions at the atomic level by using a cutoff based strategy (Fig. 1a). Here we use interactions and contacts as synonyms. For two different atoms, *i* and *j*, in the protein-protein interface, we say that *i* is in contact with *j* if *j* is inside of a sphere centered in *i* with radius *r*, called cutoff [41, 42].

In our modeling, nodes represent atoms and edges represent interactions between them. We consider only those interactions that connect atoms from different protein chains, which we name chains *A* and *B*. Therefore, we have a bipartite graph *G*(*P*,*I*,*E*) whose vertices can be segmented in 2 disjoint sets, P (nodes from *A*) and I (nodes from *B*), such that every edge in E connects a vertex in P to a vertex in I.

Nodes were labeled according to their physicochemical properties as acceptor (ACP), aromatic (ARM), donor (DON), hydrophobic (HPB), negative (NEG) or positive (POS) as in [26, 43–46]. Edges were labeled as aromatic, hydrogen bond, hydrophobic, repulsive and salt bridge, based on the physicochemical properties of their atoms and on a distance criteria. Table 1 provides the distance criteria considered for each interaction type.

**Table 1:** Physicochemical properties of atoms and distance criteria (in Å) to compute interactions

**Interaction type**	**Atom types**	**Distance**	
		MIN	MAX
Aromatic stacking	2 aromatic atoms	1.5	3.5
Hydrogen bond	1 acceptor and 1 donor atom	2.0	3.0
Hydrophobic	2 hydrophobic atoms	2.0	3.8
Repulsive	2 atoms with the same charge	2.0	6.0
Salt Bridges	2 atoms with opposite charge	2.0	6.0

Some atoms are associated with more than one label due to their ability to interact in different ways with other atoms. For instance, nitrogen atoms ND1 and NE2 in a histidine residue can be labeled aromatic, positive, donor and acceptor according to our criteria. As a consequence of this manifold aspect of node labels, multiple edges with different labels can also be attributed to a single pair of nodes. Thus, we modeled interaction graphs as multigraphs in order to capture these physicochemical aspects of protein interactions. However, its important to notice that not all node labels were attributed to the nodes. After computing interactions, only those node labels that composed interactions with other nodes were considered as final labels for the respective nodes in the graph.

Finally, for each complex (PDB entry), we computed the connected components, which serve as input data for the clustering analysis and FSM of ppiGReMLIN. So, a graph in our method corresponds to a connected component.

### Clustering analysis

A clustering analysis (Fig. 1b) takes as input a set of graphs at the atomic level representing protein-protein interfaces and organize them in similar clusters, based on physicochemical properties of its nodes and edges and graph topology, for pattern detection in the next step.

#### Graph dataset summary

**Fig. 2:**
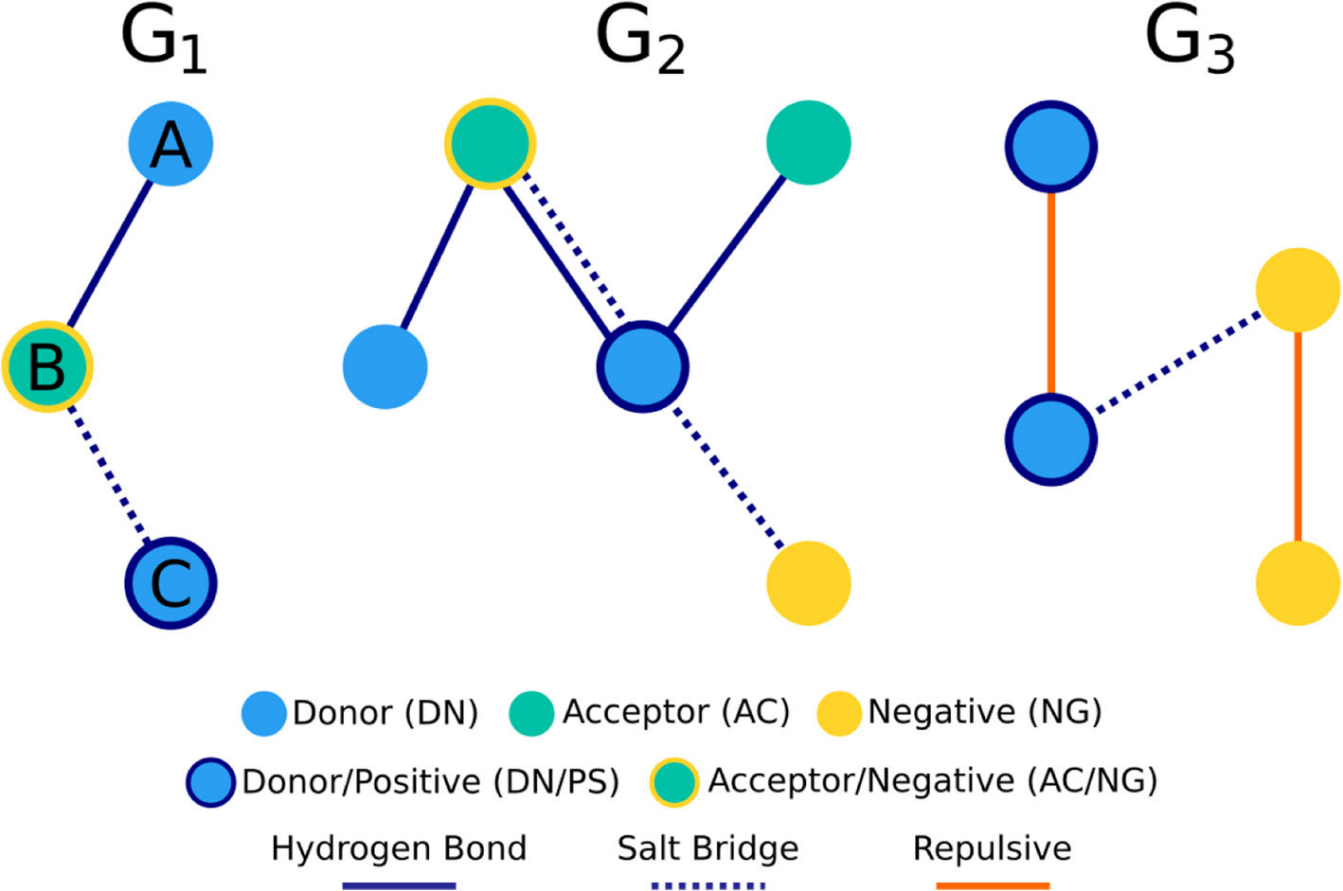
Graph sample. Graph set example for constructing the proposed counting matrix

In order to perform the clustering analysis, we propose a counting matrix, in which graphs are represented based on the labels of each of their pair of nodes. Our representation is inspired in some fingerprints that characterize small molecules [47, 48]. Suppose a graph *G*_1_ that has nodes A, B, and C, labeled as donor (DN), acceptor/negative (AC/NG), donor/positive (DN/PS) respectively, and edges A-B and B-C, representing a hydrogen bond and a salt bridge respectively, as shown in Fig. 2. To represent such graph, each pair of its nodes is labeled by joining their corresponding node labels into a single label. For example, the label DN-DN/PS represents pairs of interacting nodes where one of its atoms (DN) is a donor atom and the other one (DN/PS) is a donor/positive atom. The labels produced would be DN-AC/NG, for pair (A,B) and DN/PS-AC/NG for pair (B,C). In the resulting matrix, each row represents a graph and each column represents one of the labels produced. The counting matrix for the set of graphs in Fig. 2, which contains graph *G*_1_, is shown in Table 2.

**Table 2:** Counting matrix example

**Graph**	AC-DN/PS	AC/NG-DN	AC/NG-DN/PS	DN/PS-NG	DN/PS-DN/PS	NG-NG
*G*_1_	0	1	1	0	0	0
*G*_2_	1	1	1	1	0	0
*G*_3_	0	0	0	1	1	1

#### Dimensionality and noise reduction

Singular Value Decomposition (SVD) was used to perform dimensionality and noise reduction in ppiGReMLIN, resulting in a compact representation of the input data for clustering analysis (which we named matrix *Θ*), that can be manipulated more efficiently in terms of memory requirement and execution time [49].

SVD decomposes a rank-*lr* by *c* matrix *X* into the product *UΣV*^*T*^, where: *U* is an *r* by *l* orthogonal matrix, whose columns are the left singular vectors of *X*; *Σ* is a *l* by *l* diagonal matrix, with elements of the diagonal, denoted singular values, being positive and sorted in decreasing order; and *V* is a *c* by *l* orthogonal matrix, whose columns are the right singular vectors of *X*.

Noise reduction can be achieved by truncated SVD. In this case, a rank-*d* approximation of *X* can be obtained by retaining only: the first *d* columns of *U*, resulting in the *r* by *d* matrix *U*_*d*_; the first *d* columns and *d* rows of *Σ*, resulting in the *d* by *d* matrix *Σ*_*d*_; and the first *d* columns of *V*, resulting in the *c* by *d* matrix *V*_*d*_. It is possible to show that $X_{d} = U_{d} \Sigma _{d} V_{d}^{T}$ is the best rank-*d* approximation of *X* with regard to the root-mean-square error [50].

Dimensionality reduction can be performed by calculating the truncated SVD and taking the product *U*_*d*_*Σ*_*d*_, which results in an *r* by *d* matrix, that is an approximation of *X* with less columns. The rationale behind using *U*_*d*_*Σ*_*d*_ product to approximate *X* is that columns of *U*_*d*_ capture patterns among data objects in *X*_*d*_ [51].

The distribution of singular values was analyzed to select an appropriate *d*, reducing matrix dimensionality and keeping relevant information. The attributes of the reduced matrix are a linear combination of the attributes of the original data matrix. The number of dimensions of the reduced data matrix, *d*, was chosen considering a minimum threshold of 95% as the relative amount of the variance of the original data to be preserved [52].

#### Clustering

A clustering analysis was performed on the reduced counting matrix *Θ*, in order to group similar graphs for the subsequent step of FSM. The Spectral Clustering [53] algorithm was applied in this analysis, as it is able to group data with non-convex shapes.

The Spectral Clustering algorithm uses an internal graph representation of the data as the basis for clustering. In this graph representation, edges and their weights are conveyors of similarity information among its individuals. A similarity matrix of the data objects is used to build the graph representation. This matrix is calculated based on some measure of similarity and through different strategies, such as *k-nearest neighbors* or *ε*-*neighborhood* [54]. The clustering is then defined based on the *n* smallest eigenvalues and correspondent eigenvectors associated the with the Laplacian matrix of the graph. Here we used the scikit-learn [55] (version 0.19.2) implementation of Spectral Clustering and the *“k-nearest neighbors"* to build a graph representation of the input data. The similarity measure adopted was the euclidean distance.

One critical aspect of Spectral Clustering algorithm is the definition of a suitable number of clusters *c* and the number of neighbors *k*. It is especially hard since the selected clustering algorithm is able to identify non-convex shaped clusters in *n*-dimensional space. To circumvent this issue, we use the eigen gap heuristic [54] to support on the selection of an appropriate number of clusters by identifying the maximal difference between consecutive eigenvalues associated with the Laplacian.

#### Evaluation strategy

The appropriate number of clusters *n* given by the eigen gap heuristic was evaluated for different values of *k* (number of neighbors). These values were selected as a percentage *p* of the number of entries in each data matrix, starting at 1%, with and increment of 1%, up to 90%. Then, *k* was selected as the minimum value for which the respective similarity graph was fully connected. This is important since disconnected components in spectral clustering constitute themselves as clusters, which is not an ideal situation unless some prior information on the data indicates that [54], which is not the case.

Finally, on each dataset, evaluation of *n* and *k* was done for all rank-d matrices obtained from SVD. For each pair (*n*,*k*) obtained over each *d*, we selected the one at which *k* was minimal, within the minimal threshold of *d*, considering 95% of variance in the reduced matrix *Θ*.

### Conserved substructure mining

This block (Fig. 1c) takes as an input the groups of similar protein-protein interactions to conduct a FSM experiment which aims to detect conserved substructures in each group.

#### Subgraph mining

A FSM experiment was conducted to find conserved structural arrangements (frequent subgraphs) on protein-protein interactions for each group resulting from the clustering analysis.

In accordance with [56], considering a graph dataset *D*=*G*_0_,*G*_1_,...,*G*_*h*_,*support*(*g*) denotes the number or fraction of graphs in *D* that have *g* as a subgraph. The FSM task consists in finding any subgraph *g* whose *support*(*g*)≥*minSup* (a minimum threshold for the support). The FSM algorithm adopted was gSpan [57].

Experiments were performed with support varying from 0.1 to 1.0, with step 0.1. Resulting subgraphs went through a filter in order to extract the maximal frequent subgraphs. In a general manner, FSM algorithms are able to detect frequent patterns and point out in which input graphs such patterns were found. However, it is not possible to directly map each node/edge of a frequent pattern to a specific node/edge in an input graph. In this work, being able to perform this mapping is relevant due to the biological semantic of patterns, so that the domain specialist knows which are the relevant atoms and interactions to allow protein-protein complexes to be formed. For details, see [26, 58].

#### Subgraph-graph isomorphism

**Fig. 3:**
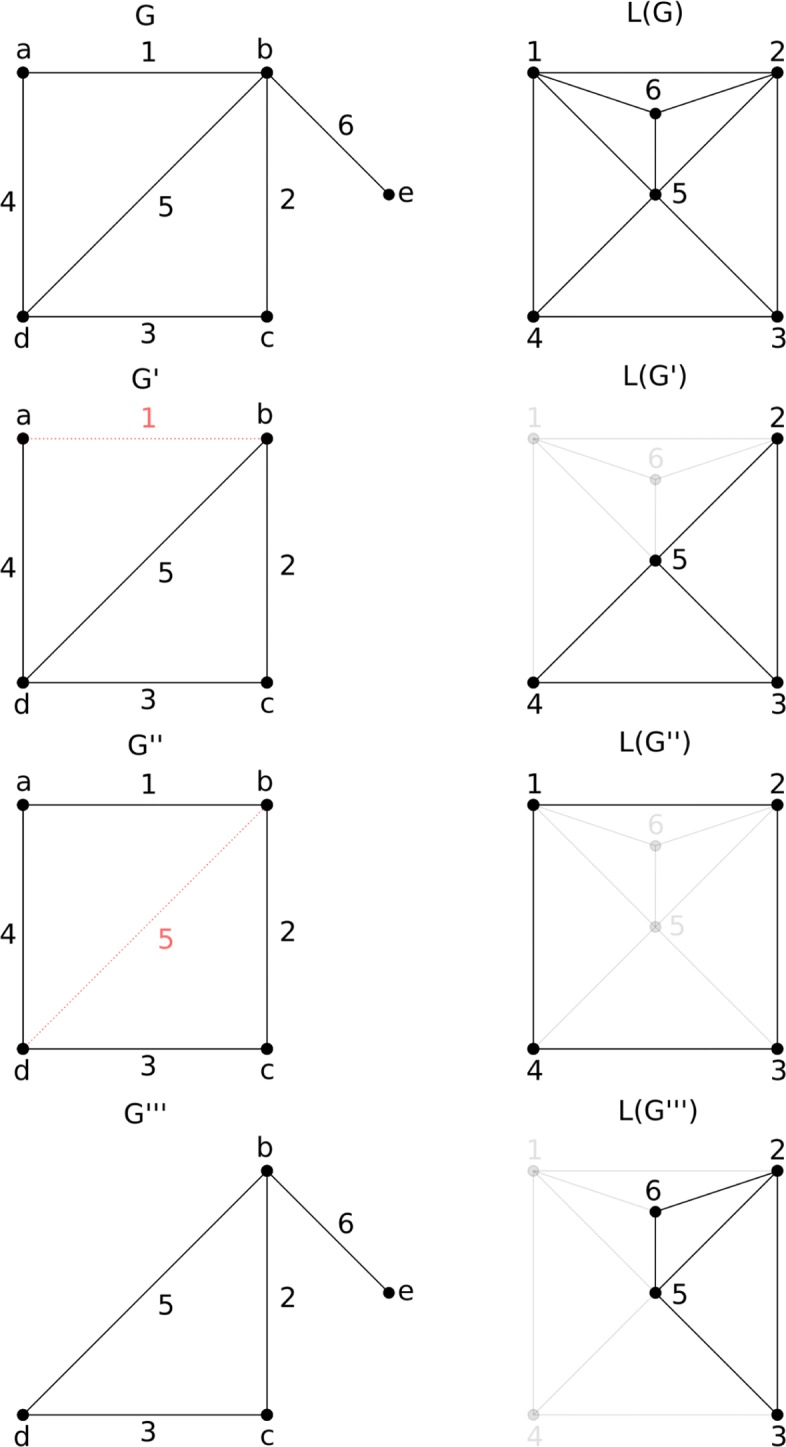
Induced subgraphs and linegraphs. Graphs on the left represent a graph *G* and a set of induced subgraphs *G*^′^,*G*^′′^,*G*^′′′^ of *G*. Red dashed edges in *G*^′^ and *G*^′′^ are the edges missing in each graph for them to become vertex-induced subgraphs of *G*. *G*^′′′^ is the only vertex-induced subgraph of *G*, as its contains all edges of its supergraph between its nodes. The linegraphs of each graph are represented on the right. Edges in a lighter color represent missing edges in *L*(*G*^′^),*L*(*G*^′′^) and *L*(*G*^′′′^), relative to the linegraph *L*(*G*). Although the original graphs are not all vertex-induced subgraphs, their line graphs are

In order to find a mapping between a subgraph *H*^′^ of a graph *H* and a graph *S*, we employed the VF2 algorithm [59]. However, implementations currently available of VF2 perform the subgraph-graph isomorphism task only for vertex-induced subgraphs. For these subgraphs, the set of edges between any pair of vertices has to be the same as the corresponding pair in their supergraphs (Fig. 3). Frequent subgraphs generated by FSM algorithm are not necessarily vertex-induced subgraphs, so we used line graphs as an alternative approach to overcome this issue.

A line graph *L*(*H*) of a simple graph *H* (Fig. 3) is obtained by associating a vertex with each edge of the original graph and connecting two vertices with an edge in the new graph if the corresponding edges of the original graph *H* have a vertex in common[60]. As stated in [61], if graphs *H* and *H*^′^ have isomorphic line graphs, then *H* and *H*^′^ are also isomorphic, with some exceptions that do not occur in our application.

Then we can simply provide VF2 with the line graphs of the original input graphs for which we want to find a mapping. However, in order to be able to use line graphs, graphs from the datasets and patterns from FSM have to be converted into simple graphs. The strategy employed at this point was to instantiate the simple graphs based on the same set of nodes of the original graphs, and connect those that are adjacent in the original graphs. Labels from multiple edges of multigraphs are joined into single labels in the new generated graphs. Next, the subgraph-graph isomorphism task is performed using node match functions so that nodes whose labels are a subset of one another may be proper evaluated as a match during the process.

## Results and discussion

To explore and confirm the ability of ppiGReMLIN to characterize and detect conserved structural arrangements on protein-protein interfaces at the atomic level we instantiated our method on two relevant datasets of protein-protein complexes. First, we show that our method can be applied to detect frequent substructures on protein-protein interfaces in large-scale. Then we compare the frequent substructures automatically detected by ppiGReMLIN to relevant residues and interactions that were experimentally determined and described in the literature.

### Clustering analysis

**Fig. 4:**
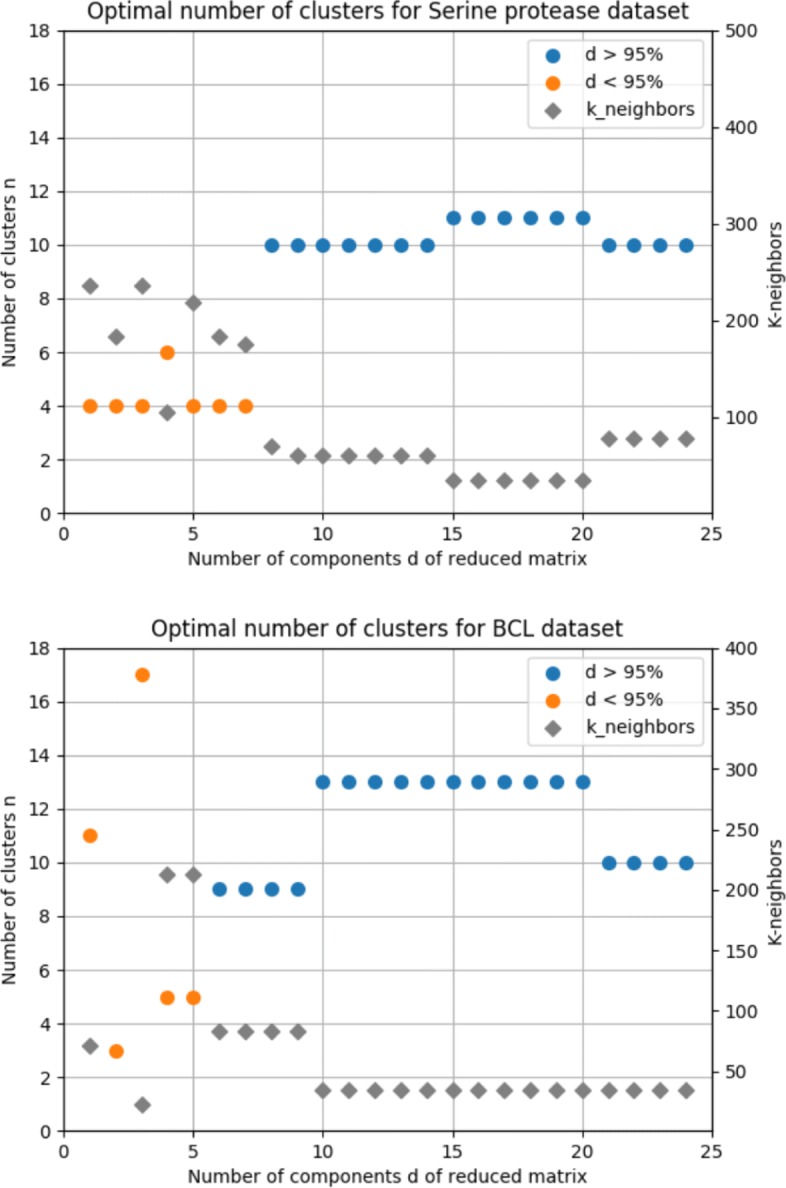
Eigen gap heuristic results. Graphs above show the results of the eigen gap heuristic for both datasets, for each reduced matrix *X*_*d*_. The number of neighbors for each experiment is also shown, represented by gray diamonds

As mentioned before, the clustering parameters were defined experimentally based on the eigen gap heuristic, which was evaluated for every instance of the count matrix regarding the number of dimensions in its reduced form. The results are summarized in Fig. 4.

For the Serine protease dataset, the number of clusters selected was *n*=11, for which the number of neighbors *k*=35 was the minimum for the experiment. For the BCL dataset, the minimum *k* for valid instances of the counting matrix (variance greater than 95%) was *k*=35, at which point the optimal number of clusters varies between 10 and 13. However, *n*=13 was selected as it occurs more frequently over a relatively larger range of *d* values (*d*∈[10,...,20]) when compared with *n*=10 (*d*∈[21,...,24]). Also, we believe the difference of the values in lower dimension instances of the data matrix is a result from the noise reduction feature offered by SVD decomposition.

### Conserved substructures mining

This section presents how proper support values were selected for each dataset and the conserved substructures detected by ppiGReMLIN in *Serine protease* and *BCL datasets*. As a general guideline, the minimum support value was set to 0.5. By increasing the support, graphs tend to be smaller in size, but more frequent in groups. Thereupon, the selection was made as a compromise between frequency of patterns and pattern size.

#### Serine protease dataset

For the serine protease dataset, at support 0.7, the largest patterns had 6 nodes and occurred in group 6. For two out of three patterns found in this group, two had real support equal to 0.83. By reducing the support value to 0.6, the number of patterns in this group went down to two, while growing in size to 7 nodes (See Supplementary materialFigure 1A). As we are interested in the most frequent substructures, we believe 0.7 to be a suitable value for minimum support, considering the small increase in pattern size.

**Fig. 5:**
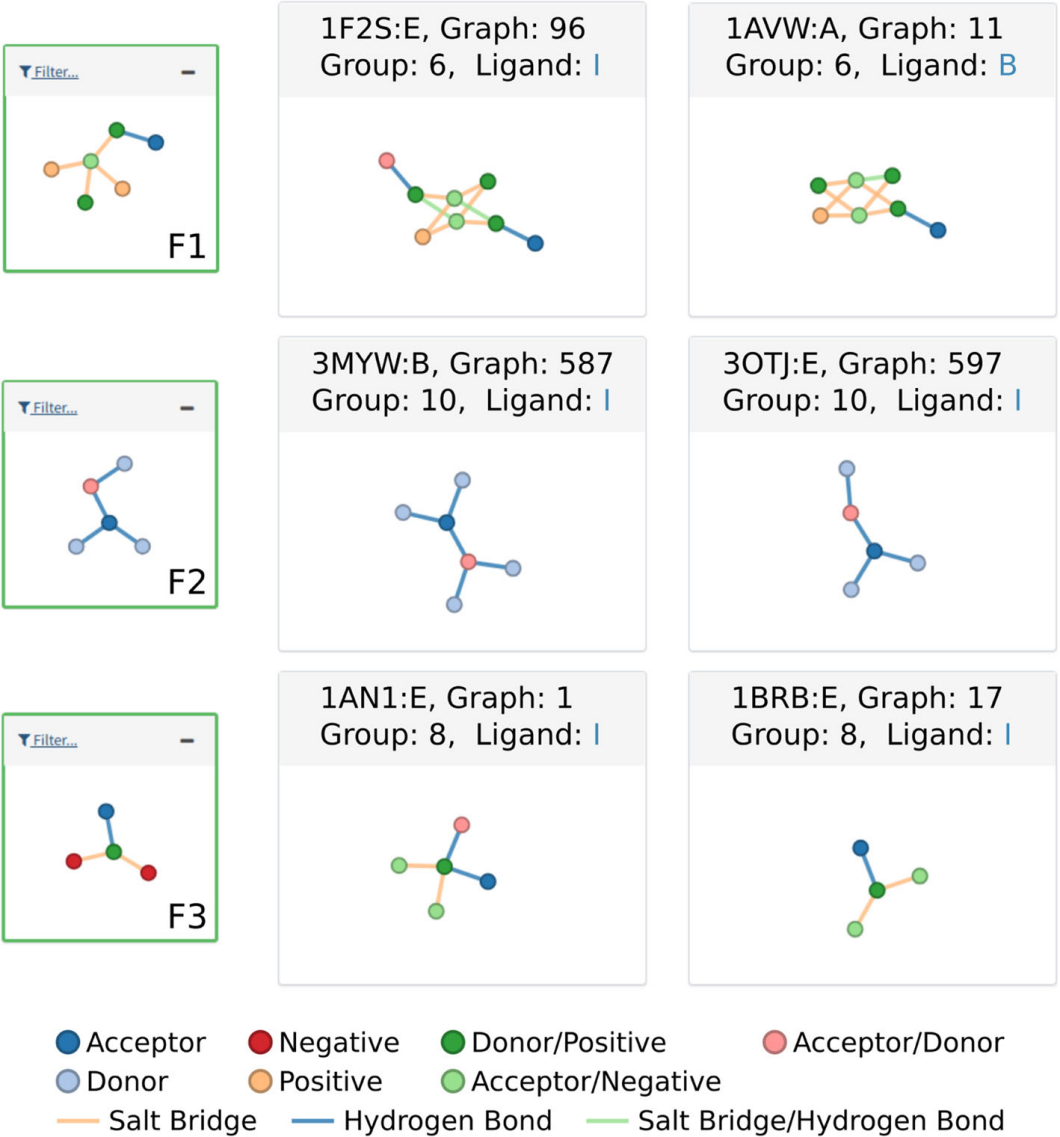
Protease conserved substructures. F1, F2 and F3 show some interesting patterns and some of their respective input graphs. Nodes and interactions in patterns might contain only a subset of the labels of its corresponding nodes in the graphs. For example, red nodes in F3 are labeled negative, meanwhile their corresponding nodes (in light green) are labeled negative and acceptor. Also, patterns may have multiple mappings to their corresponding input graphs. For example, the acceptor node from F3 (in blue) may be mapped separately to the acceptor or acceptor/donor nodes in graph 1

From this point on we discuss some of the interesting patterns found by ppiGReMLIN and summarize other groups for the selected support. Figure 5 shows the patterns we deemed most interesting and a few sample graphs in which they occur.

The largest pattern from group 6 is depicted as F1 in Fig. 5. The central vertex in this structure (4-degree vertex in the graph) represents either one of the oxygen atoms (acceptor/negative) in the carboxyl group from aspartate residues in the protease chain. The four atoms with which they interact are the nitrogen and carbon atoms (donor/positive and positive respectively) in the guanidinium group from arginine residues in the inhibitor chain. The last vertex in the structure is an oxygen atom (acceptor or acceptor/donor) from a glycine or serine residue in the protease chain. All interactions between nodes in this group occur by means of salt bridges and hydrogen bonds, as determined by the analysis of the input graphs in the group.

**Fig. 6:**
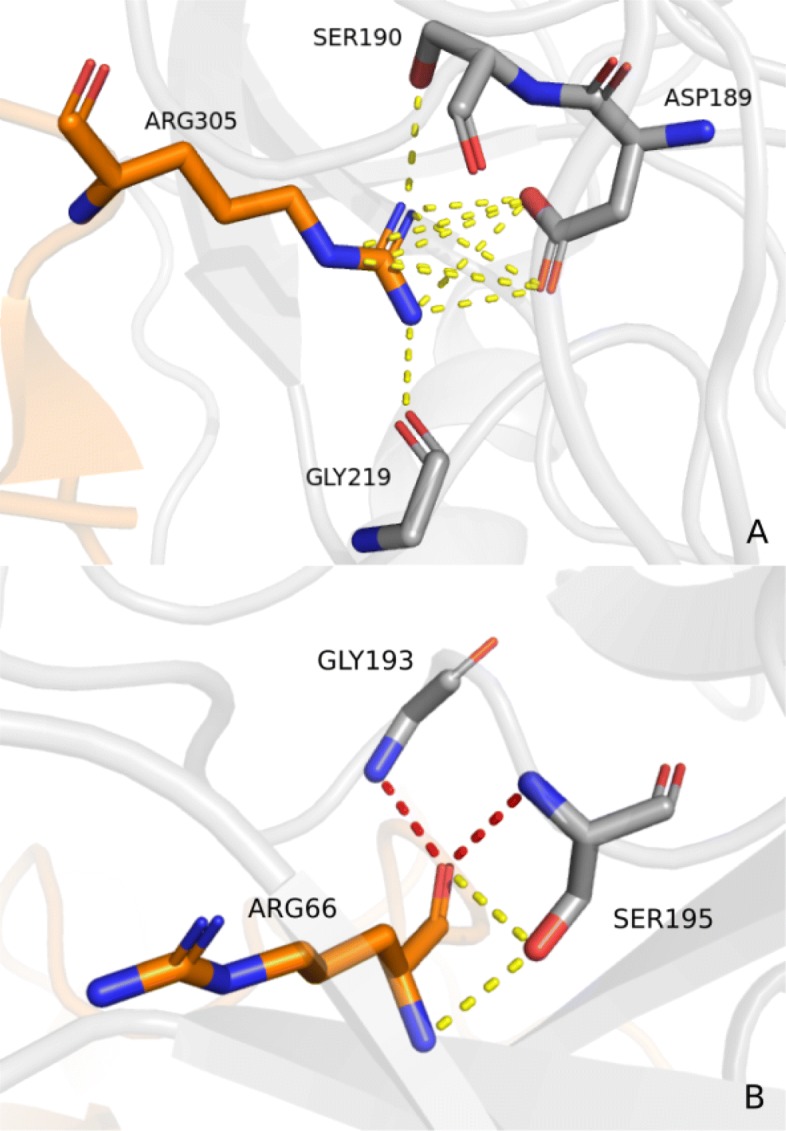
Interaction patterns presented in the context of protein structures. **a** F1 is represented in the crystal structure of the complex formed between bovine beta-trypsin and MCTI-A, a trypsin inhibitor of squash family (PDB id 1F2S). Residues in gray represent protease residues and the ones in orange represent inhibitor residues. Oxygen and nitrogen atoms are shown in red and blue respectively. **b** F2 is represented above in the kunitz type trypsin inhibitor complex with porcine trypsin (PDB id 4AN7). The oxyanion hydrogen bonds are depicted by red dashed lines, while the yellow ones represent the other hydrogen bonds that compose the F2

Regarding the residue position in the protein chain sequences for pattern F1, the majority of aspartate interacting residues (located in 32 graphs) correspond to the aspartate present in the specificity pocket (S1 pocket) of trypsin and trypsin-like proteins (See Supplementary material Figure 2). The only exception occurred in graph 24, where the interacting aspartate residue from the P1 pocket was displaced in the trypsin from the rat anionic trypsin complexed with protein ihibitor APPI (PDB id 1BRC). The serine residues (from the protease) mentioned previously were spotted in 30 of the 33 graphs where F1 was found, located right next to the aspartate P1 in the residue chain sequence. As for the glycine residues, they are positioned further in the sequence, but spatially close due the protein folding. Figure 6a shows the pattern in the crystal structure of the complex formed between bovine beta-trypsin and MCTI-A, a trypsin inhibitor of squash family (PDB id 1F2S).

Another interesting pattern, F2, is presented in Fig. 5. It was obtained from group 10 and consists of 5 nodes connected by hydrogen bonds. The central atom in the structure (3-degree vertex in the graph) represents an oxygen atom (acceptor) from the inhibitor chain interacting with two nitrogen atoms (donor) and one oxygen atom (acceptor/donor) from the protease chain. In each graph where this pattern was found, two of the atoms from the protease chain, an oxygen and a nitrogen atom, were part of the same serine residue, and the other atom, a nitrogen was from a glycine residue. The central atom in the graph represents a nitrogen atom from the inhibitor chain.

Considering pattern F2, the interacting serine residues are part of the catalytic triad in serine proteases, commonly referred as SER195, HIS57 and ASP102. (See Supplementary material Figure 2). The interaction of both the serine and the glycine residues (usually referred as GLY193) with the carbonyl atom in the substrate in structure F2 is described as an intermediate state during the process of catalysis. Figure 6b shows the interaction pattern F2 in the kunitz type trypsin inhibitor complex with porcine trypsin (PDB id 4AN7).

The last patterns of Fig. 5, represented by F3, similarly to F1, shows both oxygen atoms (acceptor negative) from the carboxyl group in aspartate residues together with an oxygen (acceptor or acceptor/donor) from a serine residue, all of them interacting with the nitrogen atoms from lysine residues in the inhibitor. Interactions in this pattern represent hydrogen bonds and salt bridges. This pattern was obtained from group 8 and occurred in all graphs in the group.

#### BCL-2 dataset

For the BCL-2 dataset, the FSM algorithm was performed with support values above 0.6 due to the size of graphs in this dataset being relatively larger (up to 30 vertices) than the ones in the *Serine protease dataset*, which generated a large number of substructures, in the order of 10^4^ to 10^5^. At support 0.6, the group with the highest number of patterns had over 50 thousand substructures, which were reduced to approximately 1,500 maximal patterns, from which only the 10 largest structures were selected for analysis.

**Fig. 7:**
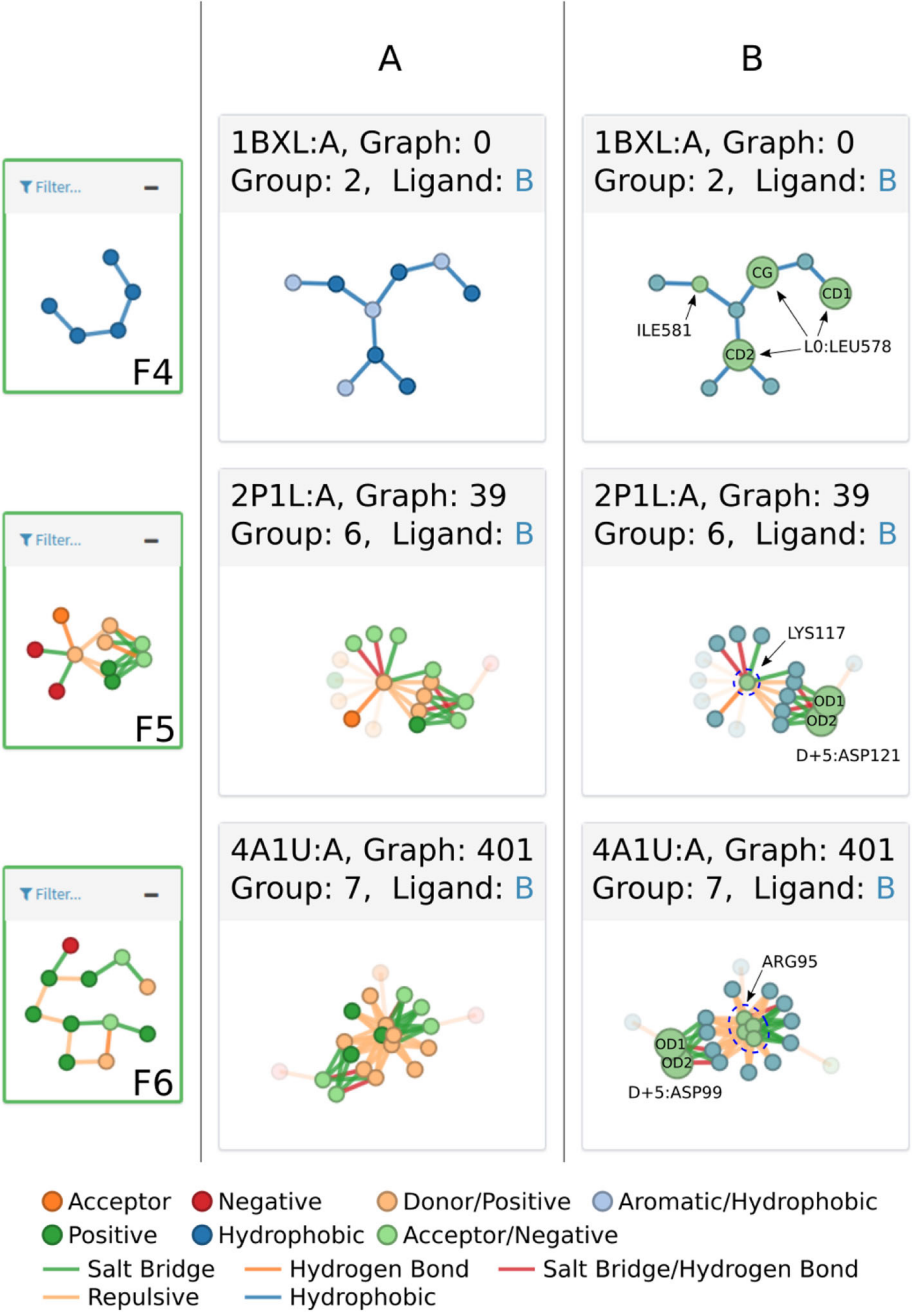
BCL-2 conserved substructures. F4, F5, F6 show some interesting patterns and some of their respective input graphs. **a** Input graphs colored according to its node labels. **b** The same input graphs with its nodes colored according to their source chain in the protein. In this example, blue nodes represent atoms from the BCL-2 antiapoptotic part of the protein complex, and green nodes represent atoms from the other protein chain in the complex. The larger green atoms atoms are part of binding hotspot residues described in literature relative to the motif LXXXXD

The support selected for the BCL-2 dataset was 0.6, since it presented the largest graphs, with 10 vertices at group 6 and 11 vertices in group 7, as opposed to 8 and 10 vertices in the same groups respectively, at support value 0.7. (See Supplementary materialFigure 1B). Figure 7 shows some of the patterns and groups deemed interesting to illustrate the ppiGReMLIN results.

Group 2 is composed of hydrophobic and aromatic atoms connected by hydrophobic and/or aromatic interactions. One of the two patterns found in this group (F4 in Fig. 7) has five hydrophobic atoms connected by hydrophobic interactions. Graph 0 (structure of Bcl-xL-Bak peptide complex PDB id 1BXL, chain A), depicted as F4-A in Fig. 7, shows one input graph in which this pattern is found.

Graphs from groups 6 and 7 are composed of acceptor/negative and donor/positive atoms connected by salt bridges, hydrogen bonds, and repulsive interactions. Patterns F5 and F6 in Fig. 7 shows one of the largest patterns from each group. One common feature in the graphs where these patterns were found is the interaction of atoms from the carboxyl group in aspartate residues in the pro-apoptotic protein chain with atoms from the guanidinium group in arginine residues in the pro-survival protein. These interactions were predominantly spotted as salt bridges. In Fig. 7b the aspartate nodes are highlighted in graph 39 (structure of the Bcl-XL:Beclin 1 complex, PDB id 2P1L, chain A) and graph 401 (crystal structure of alpha-beta-foldamer 2c in complex with Bcl-xL, PDB id 4A1U, chain A).

Another common feature in the graphs from these groups regards some interacting nodes from lysine or arginine residues in the pro-survival chain, highlighted in Fig. 7. In arginine residues, these nodes represent the atoms from the guanidinium group, while in lysine they represent the single nitrogen atom from its side chain. These atoms are found in graphs composing repulsive interactions with the arginine atoms from the pro-survival protein described before, and salt bridges or hydrogen bonds with other atoms from the pro-survival chain.

### Comparison of ppiGReMLIN with experimental patterns

We searched the literature for conserved interacting residues or structural arrangements in PPI’s for the BCL-2 and the SP datasets, which are conserved datasets regarding their sequences and structures. In order to perform a quantitative evaluation of ppiGReMLIN we used *precision* and *recall*. Borrowing the idea of these metrics from machine learning: *precision* determines the fraction of patterns that actually turns out to be relevant among those our strategy declared as relevant; *recall* measures the fraction of relevant patterns correctly recovered by our strategy. In this analysis, we consider a pattern to be relevant if the it contains at least one of the relevant residues reported by literature.

For the SP dataset, the residues considered relevant for the quantitative analysis were those experimentally determined already documented in the literature [62–64] (further details in the “Serine protease experimental patterns” section below):

- I: the reactive serine from the catalytic triad in serine proteases (usually SER195);
- II: the aspartate (usually ASP189) at the bottom of S1 pocket;
- III: the histidine, usually HIS40, that play an important role in the zymogen activation in the enzymes;
- IV: a small structure composed of two nitrogen atoms connected to a single oxygen atom by means of hydrogen bonds was used in order to target a more complex structure found in serine-proteases know as the oxyanion hole.

The numbering of residues used for the results for the SP dataset above is in agreement with PDB structure crystal structure of the complex formed between bovine beta-trypsin and mcti-a (PDB id 1F2S).

For the BCL-2 dataset, five residues in specific positions in the BH3 domain of pro-apoptotic proteins were considered as relevant for the quantitative analysis, as they play an important role in the binding of BCL-2/BH3 (further details in the “BCL-2 experimental patterns” section below):

- 1: the leucine (L0), at the beginning of the motif LXXXXD, highly conserved in BH3 domains.
- 2: the aspartate (D+5), at the end of the same motif.
- 3: residue at postion −4 (multiple possible residues)
- 4: residue at position +3 (multiple possible residues)
- 5: residue at position +7 (multiple possible residues)

Results concerning *precision* and *recall* are presented in Tables 3 and 4, for the SP and the BCL-2 datasets respectively. In each table, the results are separated by each of the groups found in the clustering step, and in the bottom line they are aggregated to give the overall perspective in the supports considered. The overall *precision* in each table is determined by the ratio between the total number of patterns pointing to at least one of the relevant residues and the total number of patterns found across all groups. The overall *recall*, on the other hand, is the ratio between the number of relevant residues mapped by ppi patterns and the total number of relevant residues.

**Table 3:** Analysis of *precision* and *recall* for the SP dataset at minimum support *0.7*

Group	Patterns	Precision	Recall	Residues
1	1	1.00	0.25	III
2	1	0.00	0.00	
3	1	1.00	0.50	II,III
4	1	0.00	0.00	
5	1	0.00	0.00	
6	3	1.00	0.25	I
7	1	1.00	0.25	IV
8	1	1.00	0.25	I
9	1	1.00	1.00	I
10	1	1.00	0.50	II,III
11	1	0.00	0.00	
All	13	0.69	1.00	I,II,III,IV

**Table 4:** Analysis of *precision* and *recall* for the BLC-2 dataset at minimum support 0.6

Group	Patterns	Precision	Recall	Residues
1	1	1.00	0.80	1,3,4,5
2	2	1.00	0.60	1,4,5
3	2	1.00	0.60	1,3,5
4	1	1.00	0.80	1,3,4,5
5	1	1.00	0.80	1,3,4,5
6	10	1.00	0.20	2
7	10	1.00	0.40	2,4
8	1	1.00	0.20	2
9	1	1.00	0.80	1,3,4,5
10	1	1.00	0.80	1,3,4,5
11	7	1.00	0.20	2
12	4	1.00	0.20	2
All	41	1.00	1.00	1,2,3,4,5

The analysis of the results shows the overall *precision* at 69% (SP) and 100% (BCL-2), while *recall* is 100% for both datasets. For the SP dataset, the lower *precision* is due to the patterns in groups 2,4,5 and 11 as they do not contain residues considered relevant documented in the literature. However, we were able to find all the relevant structures in the ppiGReMLIN patterns, as shown by the overall *recall* (100%). Interestingly, some of the test structures were identified alone in specific groups, e.g., III in group 1, IV in group 7, and I in groups 6,8 and 9, for the SP dataset; and structure 2 in groups 6,8,11 and 12, for the BCL-2 dataset. This shows how ppiGReMLIN not only can identify interaction patterns, but also is able to single out some relevant interactions with specific residues in PPI’s, which also demonstrates the importance of the clustering step in the strategy workflow.

The fact that we did not find patterns documented in the literature in groups 2,4,5 and 11 does not mean that there are no relevant binding patterns in these groups. This only means that the patterns found are not documented in the literature. There is a possibility that such patterns are relevant and related to protein-protein interactions. Thinking of proteins as a network of interaction between residues, there are very few residues at the binding site and even less residues at the active site. Nonetheless, there are a number of residues that can be important in this network of residues to help, for example, stabilize the residues that are at the binding site. We believe that the patterns in the groups 2,4,5 and 11 should be further investigated to understand whether they are relevant.

#### Serine protease experimental patterns

**Fig. 8:**
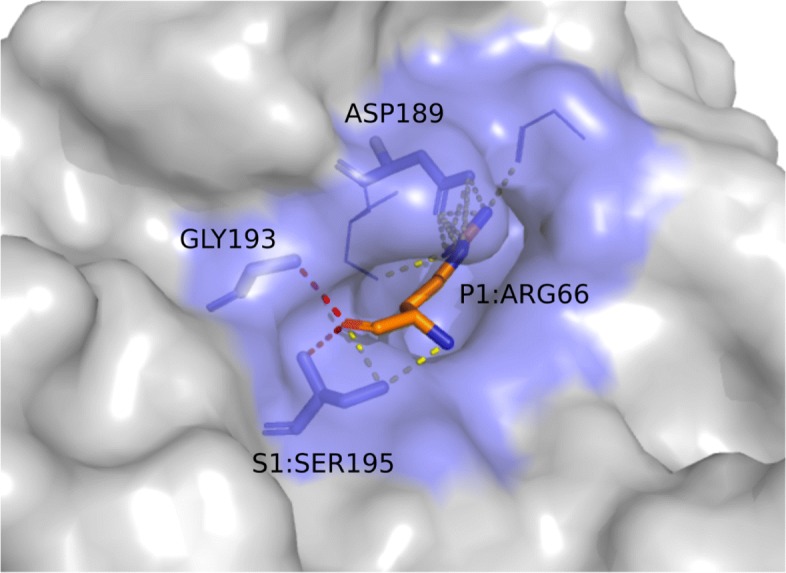
Interacting residues at trypsin *S*1 pocket. The *S*1 pocket is shown above in the kunitz type trypsin inhibitor complex with porcine trypsin (PDB id 4AN7). The protease surface is depicted in gray with the *S*1 pocket highlighted in blue. Residue at position S1 from the protease and P1 from the inhibitor are indicated along with ASP189 and GLY193, which are relevant residues as described in literature. Pattern F2 is represented above in the interactions of the P1 residue with ASP189, along with other two neighboring residues. F1 is represented by the interaction of residues in position P1, S1 and GLY193, with the hydrogen bonds from an oxyanion hole depicted in red dashed lines

According to Perona and Craik [64], position 189 located at the base of the S1 pocket (Fig. 8), is highly conserved as an aspartate in enzymes with trypsin-like specificity towards substrates that contain arginine and lysine. The role of the negatively charged ASP189 in binding and catalysis has been addressed in many studies [65–68]. In [64], this interaction is said to occur by means of hydrogen bonds mediated by water molecules for substrates with lysine at position P1. For substrates with arginine residues, it is also possible that these interactions are formed as salt bridges. Our results from patterns in groups 6 and 10, described in the previous section, shows these interactions predominantly as salt bridges, even for lysine residues. The reason for this lies in the criteria employed in the contact prospecting phase, in which hydrogen bonds mediated by water were not considered, only direct hydrogen bonding between atoms were taken in account. Thus, we would not expect to find such interactions. However, due to the overlapping distance range of both hydrogen bonds and salt bridges, and physicochemical properties of interacting atoms, the strategy was able to spot the residues as relevant in the protein-protein interaction interface.

Another interaction mentioned in the literature [64] was found in group 10 (described by F2), and in many graphs in group 3. The structure contains an interaction known as the *oxyanion hole*, composed of two amide nitrogen atoms, usually from SER195 and GLY193, in the protease chain interacting with a carbonyl oxygen atom in the inhibitor chain by means of hydrogen bonds. The role of the *oxyanion hole* in the catalysis is to stabilize the negative charge in the carbonyl group after it becomes a tetrahedral intermediate, i.e, the double bond between its carbon and oxygen atoms becomes a single-bond [69]. The structure is depicted in Fig. 6b.

Additionally, the GLY193 residue, which is highly conserved in serine proteases, is frequent in the patterns described above. According to [70], it makes a crucial contribution to substrate binding in both the enzyme ground and transition states during catalysis.

#### BCL-2 experimental patterns

**Fig. 9:**
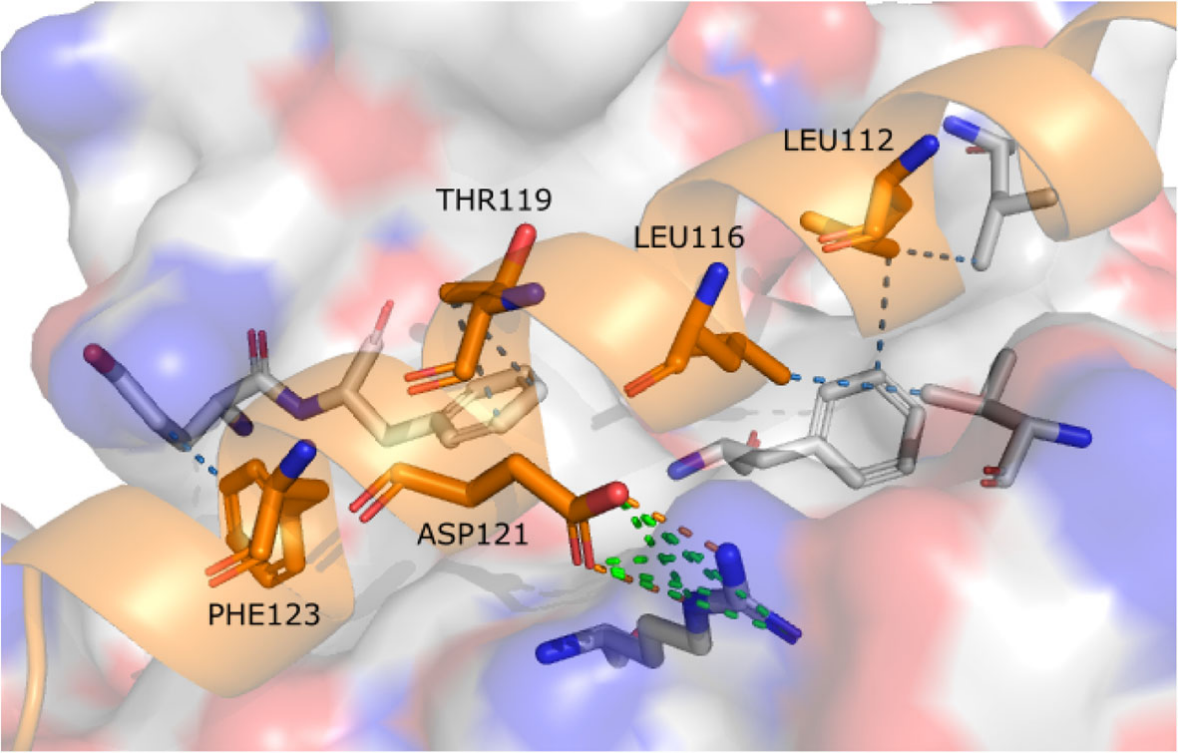
Critical interactions for the BCL-2/BH3 binding. Critical residue interactions are shown in the structure of the Bcl-XL:Beclin 1 complex (PDB id 2P1L). The Beclin 1 chain, a BH3 only protein, is shown in orange in the hydrophobic groove of Bcl-xL, an anti apoptotic protein. Hydrophobic interactions are depicted by dashed lines in blue, salt bridges in green, and hydrogen bonds in orange. The five interacting residues from BH3 chain represent those found in patterns and in literature

The molecular recognition between pro-apoptotic and anti-apoptotic members of the BCL-2 family is driven by the ability of the amphipathic BH3 *α*-helix of regulators to be accommodated in the hydrophobic groove formed by the four BH domains in the suppressors [71] (Fig. 9). This interaction paradigm is shared between both BH3-only (activators) and multi-domain (effectors) proteins of pro-apoptotic BCL-2 members, which act competitively towards pro-survival members in order to regulate apoptosis. Natively, effectors are complexed with anti-apoptotic members in healthy cells, being displaced by activators in response to pro-apoptotic signals, which ultimately culminates in cell death [72].

**Fig. 10:**
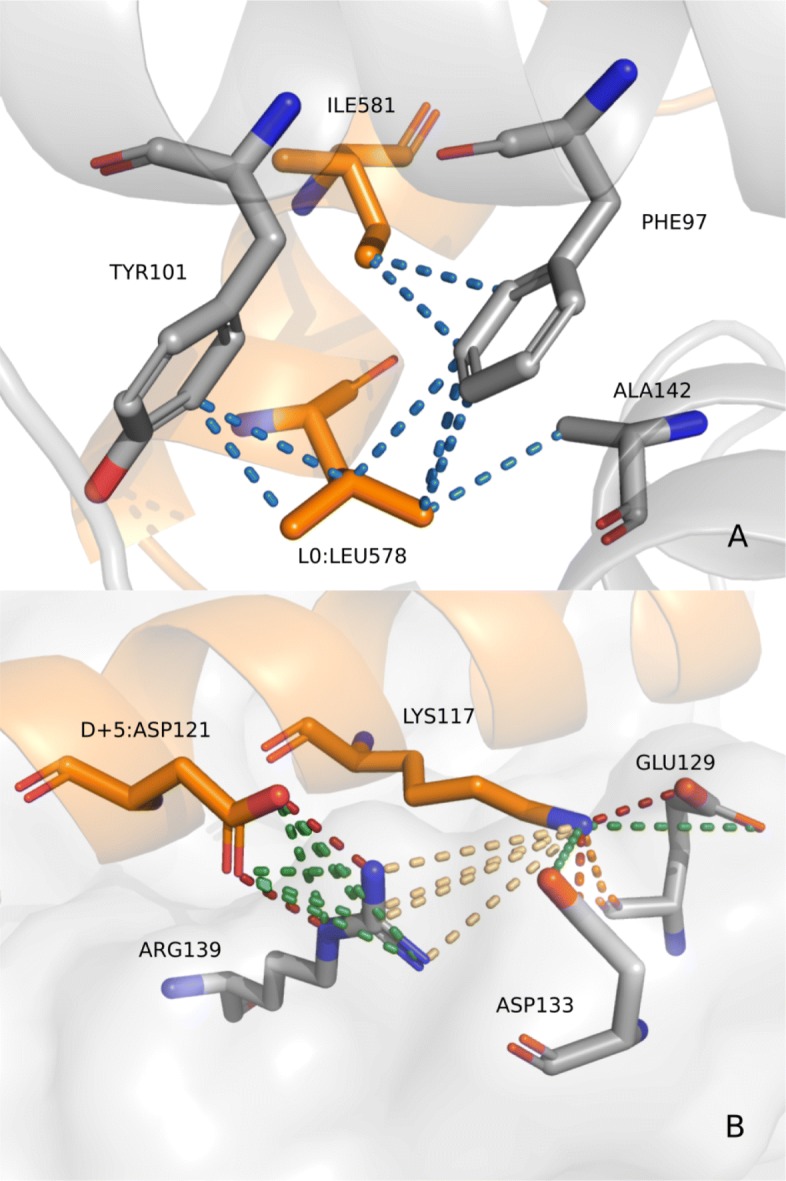
Interaction patterns presented in the context of protein structures. **a** Graph 0, containing F4, is represented in the structure of Bcl-xL-Bak peptide complex (PDB id 1BXL). Residues in gray represent protease residues and the ones in orange represent inhibitor residues. Oxygen and nitrogen atoms are shown in red and blue respectively. Residue *L*0 from motif LXXXXD in BH3 pro-apoptosis regulator Bak is shown here as LEU578 (**b**) Graph 39, containing F5, is represented above in the crystal structure of alpha-beta-foldamer 2c in complex with Bcl-xL (PDB id 4A1U). Salt bridges are depicted in green, hydrogen bonds in orange, and repulsive interactions in light yellow. Red dashed lines represent edges where interactions are described both as hydrogen bonds and salt bridges. Residue at position D+5 is highlighted here as ASP121

The BH3 domain of both effectors and activators, typically about 20 amino acids in length, is characterized by the presence of the motif LXXXXD. While interactions may occur along the BH3 chain, only a few of its residues have been characterized as critical to the specificity of binding towards suppressors. Apart from the leucine (L0), flanking the motif LXXXXD, hydrophobic residues at positions −4, +3 and +7, represent binding hotspots in the molecular recognition [71]. The hydrophobic interactions promoted by these residues were found in patterns from groups 1,2,3,4,5 and also in patterns from group 9. Graph 0, in Fig. 7b, highlights atoms from the leucine L0 and from isoleucine at position +3 interacting with other atoms in the suppressor chain by means of hydrophobic interactions, and atoms from isoleucine at position +3. The structure can also be visualized in Fig. 10a.

The aspartate (D+5) in the motif LXXXXD, is the last of the critical residues mentioned in literature, described to interact with atoms from suppressor residues by means of hydrogen bonds or salt bridges [71]. This interaction was found in patterns from groups 6,7,8 and in patterns from groups 10,11,12. F5 and F6, in Fig. 7b, highlight atoms from aspartate D+5 interacting with atoms in the guanidinium group from arginine residues in the suppressor chain. Figure 10b shows a visualization of input graph 401, one structure on which F5 was found, with aspartate D+5 (here as ASP121) highlighted.

Interestingly, on the same structures described above, atoms from residues at position +1 were also found composing interactions. These residues were either arginine or lysine. F5 and F6 in Fig. 7 highlight these residues, which are shown as LYS117 in graph 39 and ARG95 in graph 401. Although this position is not critical for the binding of BH3 domains and BCL-2 anti-apoptotic proteins, their role in the binding affinity cannot be overlooked. Boersma and colleagues[73] demonstrated that substitution for glutamine inhibited binding of pro-apoptotic Bim-BH3 with anti-apoptotic proteins Bcl-xL and Mcl-1, while remaining unchanged for uncharged residues. Furthermore, Dutta and colleagues [72] showed different binding profiles for mutated Bim-BH3 towards Bcl-xL and Mcl-1. Substitutions with negatively charged residues were tolerated for binding to Mcl-1, whereas for Bcl-xL, it resulted in low binding affinity.

## Conclusions

This work proposed a graph based strategy to detect conserved structural arrangements on protein-protein interface, which we named ppiGReMLIN. In this method, protein-protein interfaces are modeled as graphs at the atomic level. Atoms and non-covalent interactions are represented by nodes and edges respectively, which are labeled according to their physicochemical properties and distance criteria. The resulting set of graphs is segmented in similar clusters that serve as the input for a FSM, revealing substructures that are conserved across the whole dataset of protein-protein interactions. Our method does not rely on sequence alignment nor structural superimposition, and can be used in large-scale datasets of protein-protein interactions.

Results show the method is effective in finding conserved structures described in the literature in an automatic fashion. At the supports chosen for each of our main datasets, *precision* range from 69% up to 100%, with *recall* of 100%, considering the most relevant structures found in literature. For instance, from the serine protease dataset, patterns were found with support higher than 70%. Among these, structures such as the SER195 and GLY193 oxyanion, an important interaction in intermediate states of catalysis, and the ASP189 interactions in the binding specificity pocket of trypsin, were detected in more than a single cluster. Relevant interacting residues were also found for the BCL-2/BH3 dataset with support higher then 60%. Residues at critical positions relative to the motif LXXXXD, which characterizes the BH3 domain of apoptotic regulators, were found in many patterns across different groups. Additionally, relevant residues were also found in non-critical positions in BH3 domains, that influence the binding affinity of BCL-2 members.

As future work, we plan to calculate water mediated interactions by considering structural water molecules. Also, we intend to make ppiGReMLIN available as a web-server and as web services, which will allow our method to be easily accessible for users and to be included in automatic pipelines.

## Supplementary information

**Additional file 1** Supplementary Material.

## Data Availability

The source code and datasets are available at: https://github.com/ppigremlin/ppiGremlin. The prototype tool that allows visualization of results is available at: https://ppigremlin.github.io/.

## Abbreviations

AG: Anticarcia gemmatalis
FSM: Frequent subgraph mining
KTI: Kunitz trypsin inhibtor
PDB: Protein data bank
PPI: Protein-protein interactions
RNA: Ribonucleic acid
SVD: Singular value decomposition

## References

[1] Machleidt T, Woodroofe CC, Schwinn MK, Mendez J, Robers MB, Zimmerman K, Otto P, Daniels DL, Kirkland TA, Wood KV (2015). Nanobret– a novel bret platform for the analysis of protein–protein interactions. ACS Chem Biol.

[2] Braun P, Gingras A-C (2012). History of protein–protein interactions: From egg-white to complex networks. Proteomics.

[3] Das J, Yu H (2012). Hint: High-quality protein interactomes and their applications in understanding human disease. BMC Syst Biol.

[4] Chatr-Aryamontri A, Oughtred R, Boucher L, Rust J, Chang C, Kolas NK, O’Donnell L, Oster S, Theesfeld C, Sellam A (2017). The biogrid interaction database: 2017 update. Nucleic Acids Res.

[5] Alonso-López D, Campos-Laborie FJ, Gutiérrez MA, Lambourne L, Calderwood MA, Vidal M, De Las Rivas J. Apid database: redefining protein–protein interaction experimental evidences and binary interactomes. Database. 2019; 2019. 10.1093/database/baz005. 10.1093/database/baz005 PMC6354026 30715274

[6] Kamburov A, Stelzl U, Lehrach H, Herwig R (2012). The consensuspathdb interaction database: 2013 update. Nucleic Acids Res.

[7] Wong AK, Krishnan A, Yao V, Tadych A, Troyanskaya OG (2015). Imp 2.0: a multi-species functional genomics portal for integration, visualization and prediction of protein functions and networks. Nucleic Acids Res.

[8] Kotlyar M, Pastrello C, Sheahan N, Jurisica I (2015). Integrated interactions database: tissue-specific view of the human and model organism interactomes. Nucleic Acids Res.

[9] Szklarczyk D, Morris JH, Cook H, Kuhn M, Wyder S, Simonovic M, Santos A, Doncheva NT, Roth A, Bork P, et al.The string database in 2017: quality-controlled protein–protein association networks, made broadly accessible. Nucleic Acids Res. 2016:937. 10.1093/nar/gkw937. 10.1093/nar/gkw937 PMC5210637 27924014

[10] Ogris C, Guala D, Kaduk M, Sonnhammer EL (2017). Funcoup 4: new species, data, and visualization. Nucleic Acids Res.

[11] Franz M, Rodriguez H, Lopes C, Zuberi K, Montojo J, Bader GD, Morris Q (2018). Genemania update 2018. Nucleic Acids Res.

[12] Li T, Wernersson R, Hansen RB, Horn H, Mercer J, Slodkowicz G, Workman CT, Rigina O, Rapacki K, Stærfeldt HH (2017). A scored human protein–protein interaction network to catalyze genomic interpretation. Nat Methods.

[13] Stumpf MP, Thorne T, de Silva E, Stewart R, An HJ, Lappe M, Wiuf C (2008). Estimating the size of the human interactome. Proc Natl Acad Sci.

[14] Venkatesan K, Rual J-F, Vazquez A, Stelzl U, Lemmens I, Hirozane-Kishikawa T, Hao T, Zenkner M, Xin X, Goh K-I (2009). An empirical framework for binary interactome mapping. Nat Methods.

[15] Blundell TL, Sibanda BL, Montalvão RW, Brewerton S, Chelliah V, Worth CL, Harmer NJ, Davies O, Burke D (2006). Structural biology and bioinformatics in drug design: opportunities and challenges for target identification and lead discovery. Phil Trans R Soc B Biol Sci.

[16] Meireles LM, Domling AS, Camacho CJ (2010). Anchor: a web server and database for analysis of protein–protein interaction binding pockets for drug discovery. Nucleic Acids Res.

[17] Nevola L, Giralt E (2015). Modulating protein–protein interactions: the potential of peptides. Chem Commun.

[18] Jubb H, Blundell TL, Ascher DB (2015). Flexibility and small pockets at protein–protein interfaces: new insights into druggability. Prog Biophys Mol Biol.

[19] Scott DE, Bayly AR, Abell C, Skidmore J (2016). Small molecules, big targets: drug discovery faces the protein–protein interaction challenge. Nat Rev Drug Discov.

[20] Pelay-Gimeno M, Glas A, Koch O, Grossmann TN (2015). Structure-based design of inhibitors of protein–protein interactions: Mimicking peptide binding epitopes. Angew Chem Int Ed.

[21] Fuller JC, Burgoyne NJ, Jackson RM (2009). Predicting druggable binding sites at the protein–protein interface. Drug Discovery Today.

[22] Craik DJ, Fairlie DP, Liras S, Price D (2013). The future of peptide-based drugs. Chem Biol Drug Des.

[23] Khashan R, Zheng W, Tropsha A (2012). Scoring protein interaction decoys using exposed residues (spider): a novel multibody interaction scoring function based on frequent geometric patterns of interfacial residues. Proteins Struct Funct Bioinforma.

[24] Morozova N, Allers J, Myers J, Shamoo Y (2006). Protein–rna interactions: exploring binding patterns with a three-dimensional superposition analysis of high resolution structures. Bioinformatics.

[25] Melo R, Ribeiro C, Murray C, Veloso C, da Silveira C, Neshich G, Meira Jr W, Carceroni R, Santoro M (2007). Finding protein-protein interaction patterns by contact map matching. Genet. Mol. Res.

[26] Santana CA, Cerqueira FR, da Silveira CH, Fassio AV, de Melo-Minardi RC, Silveira S. d. A.Gremlin: a graph mining strategy to infer protein-ligand interaction patterns. In: Bioinformatics and Bioengineering (BIBE), 2016 IEEE 16th International Conference On. IEEE: 2016. p. 28–35. 10.1109/bibe.2016.48.

[27] Pilon FM, Silva C. d. R., Visôtto LE, Barros R. d. A., da Silva Júnior NR, Campos WG, de Almeida Oliveira MG (2017). Purification and characterization of trypsin produced by gut bacteria from anticarsia gemmatalis. Arch Insect Biochem Physiol.

[28] Patarroyo-Vargas AM, Merino-Cabrera YB, Zanuncio JC, Rocha F, Campos WG, de Almeida O, Maria G (2017). Kinetic characterization of anticarsia gemmatalis digestive serine-proteases and the inhibitory effect of synthetic peptides. Protein Pept Lett.

[29] Vianna U, Pratissoli D, Zanuncio J, Alencar J, Zinger FD (2011). Espécies e/ou linhagens de trichogramma spp. (hymenoptera: Trochogrammatidae) para o controle de anticarsia gemmatalis (lepidoptera: Noctuidae). Arquivos do Instituto Biológico.

[30] Scott IM, Thaler JS, Scott JG (2010). Response of a generalist herbivore trichoplusia ni to jasmonate-mediated induced defense in tomato. J Chem Ecol.

[31] Wielkopolan B, Walczak F, Podleśny A, Nawrot R, Obrępalska-Stęplowska A (2015). Identification and partial characterization of proteases in larval preparations of the cereal leaf beetle (oulema melanopus, chrysomelidae, coleoptera). Arch Insect Biochem Physiol.

[32] GenBank Internet, Bethesda MD. National Library of Medicine (US), National Center for Biotechnology Information. 1982. https://www.ncbi.nlm.nih.gov/nuccore/JX898746.1.

[33] Benson DA, Karsch-Mizrachi I, Lipman DJ, Ostell J, Wheeler DL (2008). Genbank. Nucleic Acids Res.

[34] Berman HM, Westbrook J, Feng Z, Gilliland G, Bhat TN, Weissig H, Shindyalov IN, Bourne PE (2000). The protein data bank. Nucleic Acids Res.

[35] Krajewski S, Tanaka S, Takayama S, Schibler MJ, Fenton W, Reed JC (1993). Investigation of the subcellular distribution of the bcl-2 oncoprotein: residence in the nuclear envelope, endoplasmic reticulum, and outer mitochondrial membranes. Cancer Res.

[36] Cai J, Yang J, Jones D (1998). Mitochondrial control of apoptosis: the role of cytochrome c. Biochim Biophys Acta (BBA)-Bioenerg.

[37] Opferman JT, Kothari A (2018). Anti-apoptotic bcl-2 family members in development. Cell Death Differ.

[38] Delbridge AR, Valente LJ, Strasser A (2012). The role of the apoptotic machinery in tumor suppression. Cold Spring Harbor Perspect Biol.

[39] Delbridge A, Strasser A (2015). The bcl-2 protein family, bh3-mimetics and cancer therapy. Cell Death Differ.

[40] Hanahan D, Weinberg RA (2011). Hallmarks of cancer: the next generation. Cell.

[41] da Silveira CH, Pires DE, Minardi RC, Ribeiro C, Veloso CJ, Lopes JC, Meira W, Neshich G, Ramos CH, Habesch R (2009). Protein cutoff scanning: A comparative analysis of cutoff dependent and cutoff free methods for prospecting contacts in proteins. Proteins Struct Funct Bioinforma.

[42] Martins PM, Mayrink VD, de A Silveira S, da Silveira CH, de Lima LH, de Melo-Minardi RC. How to compute protein residue contacts more accurately? In: Proceedings of the 33rd Annual ACM Symposium on Applied Computing. ACM: 2018. p. 60–67. 10.1145/3167132.3167136.

[43] Gonçalves-Almeida VM, Pires DE, de Melo-Minardi RC, da Silveira CH, Meira W, Santoro MM (2011). Hydropace: understanding and predicting cross-inhibition in serine proteases through hydrophobic patch centroids. Bioinformatics.

[44] Silveira SA, Fassio AV, Gonçalves-Almeida VM, de Lima EB, Barcelos YT, Aburjaile FF, Rodrigues LM, Meira Jr W, de Melo-Minardi RC. Vermont: Visualizing mutations and their effects on protein physicochemical and topological property conservation. In: BMC Proceedings, vol. 8. BioMed Central: 2014. p. 4. 10.1186/1753-6561-8-s2-s4. 10.1186/1753-6561-8-S2-S4 PMC4155615 25237391

[45] Fassio AV, Martins PM, Guimarães S. d. S., Junior SS, Ribeiro VS, de Melo-Minardi RC, Silveira S. d. A. (2017). Vermont: a multi-perspective visual interactive platform for mutational analysis. BMC Bioinformatics.

[46] Fassio AV, Santana CA, Cerqueira FR, da Silveira CH, Romanelli JP, de Melo-Minardi RC, Silveira S. d. A.An interactive strategy to visualize common subgraphs in protein-ligand interaction. In: International Conference on Bioinformatics and Biomedical Engineering. Springer: 2018. p. 383–94. 10.1007/978-3-319-78723-7_33.

[47] Liu K, Feng J, Young SS (2005). Powermv: a software environment for molecular viewing, descriptor generation, data analysis and hit evaluation. J Chem Inf Model.

[48] Cereto-Massagué A, Ojeda MJ, Valls C, Mulero M, Garcia-Vallvé S, Pujadas G (2015). Molecular fingerprint similarity search in virtual screening. Methods.

[49] Eldén L (2006). Numerical linear algebra in data mining. Acta Numerica.

[50] Leskovec J, Rajaraman A, Ullman J (2014). Mining of Massive Datasets, Chapter 11: Dimensionality Reduction.

[51] Tan P-N (2006). Introduction to Data Mining.

[52] Zaki MJ, Meira Jr W, Meira W (2014). Data Mining and Analysis: Fundamental Concepts and Algorithms.

[53] Ng AY, Jordan MI, Weiss Y (2001). On spectral clustering: Analysis and an algorithm. Proceedings of the 14th International Conference on Neural Information Processing Systems: Natural and Synthetic, NIPS’01.

[54] Von Luxburg U (2007). A tutorial on spectral clustering. Stat Computing.

[55] Pedregosa F, Varoquaux G, Gramfort A, Michel V, Thirion B, Grisel O, Blondel M, Prettenhofer P, Weiss R, Dubourg V, Vanderplas J, Passos A, Cournapeau D, Brucher M, Perrot M, Duchesnay E (2011). Scikit-learn: Machine learning in Python. J Mach Learn Res.

[56] Jiang C, Coenen F, Zito M (2013). A survey of frequent subgraph mining algorithms. Knowl Eng Rev.

[57] Yan X, Han J. gspan: Graph-based substructure pattern mining. In: Data Mining, 2002. ICDM 2003. Proceedings. 2002 IEEE International Conference On. IEEE: 2002. p. 721–4. 10.1109/icdm.2002.1184038.

[58] Fassio AV, Santana CA, Cerqueira FR, Romanelli JPR, da Silveira CH, de Melo-Minardi RC, Silveira SA. An interactive strategy to visualize common subgraphs in protein-ligand interaction. In: Bioinformatics and Biomedical Engineering (IWBBIO), 6th International Work-Conference On: 2018. Paper accepted. 10.1007/978-3-319-78723-7_33.

[59] Cordella LP, Foggia P, Sansone C, Vento M (2004). A (sub) graph isomorphism algorithm for matching large graphs. IEEE Trans Pattern Anal Mach Intell.

[60] Gross JL, Yellen J (2005). Graph Theory and Its Applications.

[61] Harary F (1969). Graph Theory. Addison-Wesley series in mathematics.

[62] Steitz TA, Shulman RG (1982). Crystallographic and nmr studies of the serine proteases. Annu Rev Biophys Bioeng.

[63] Markley JL, Ibanez IB (1978). Zymogen activation in serine proteinases. proton magnetic resonance ph titration studies of the two histidines of bovine chymotrypsinogen a and chymotrypsin a. alpha. Biochemistry.

[64] Perona JJ, Craik CS (1995). Structural basis of substrate specificity in the serine proteases. Protein Sci.

[65] Graf L, Craik CS, Patthy A, Roczniak S, Fletterick RJ, Rutter WJ (1987). Selective alteration of substrate specificity by replacement of aspartic acid-189 with lysine in the binding pocket of trypsin. Biochemistry.

[66] Gráf L, Jancso A, Szilágyi L, Hegyi G, Pintér K, Náray-Szabó G, Hepp J, Medzihradszky K, Rutter WJ (1988). Electrostatic complementarity within the substrate-binding pocket of trypsin. Proc Natl Acad Sci.

[67] Perona JJ, Tsu CA, McGrath ME, Craik CS, Fletterick RJ (1993). Relocating a negative charge in the binding pocket of trypsin. J Mol Biol.

[68] Evnin LB, Vásquez JR, Craik CS (1990). Substrate specificity of trypsin investigated by using a genetic selection. Proc Natl Acad Sci.

[69] Zakharova E, Horvath MP, Goldenberg DP (2009). Structure of a serine protease poised to resynthesize a peptide bond. Proc Natl Acad Sci.

[70] Bobofchak KM, Pineda AO, Mathews FS, Di Cera E (2005). Energetic and structural consequences of perturbing gly-193 in the oxyanion hole of serine proteases. J Biol Chem.

[71] Bhat V, Olenick MB, Schuchardt BJ, Mikles DC, McDonald CB, Farooq A (2013). Biophysical basis of the promiscuous binding of b-cell lymphoma protein 2 apoptotic repressor to bh3 ligands. J Mol Recog.

[72] Dutta S, Gullá S, Chen TS, Fire E, Grant RA, Keating AE (2010). Determinants of bh3 binding specificity for mcl-1 versus bcl-xl. J Mol Biol.

[73] Boersma MD, Sadowsky JD, Tomita YA, Gellman SH (2008). Hydrophile scanning as a complement to alanine scanning for exploring and manipulating protein–protein recognition: application to the bim bh3 domain. Protein Sci.

