# Supplementary material for: ppiGReMLIN: a graph mining based detection of conserved structural arrangements in protein-protein interfaces
Source: BMC Bioinformatics. 2020 Apr 15;21:143. doi: 10.1186/s12859-020-3474-1 (PMC7158050; doi:10.1186/s12859-020-3474-1)
Supplement: Supplementary file 1 — Additional file 1 Supplementary Material. [file 12859_2020_3474_MOESM1_ESM.pdf]

PPIGREMLIN: A GRAPH MINING BASED DETECTION OF CONSERVED STRUCTURAL ARRANGEMENTS IN PROTEIN-PROTEIN INTERFACES - SUPPLEMENTARY MATERIAL

Results and Discussion

Support	0.6											0.7										
Graph Size	1	2	3	4	5	6	7	8	9	10	11	1	2	3	4	5	6	7	8	9	10	11
2	1			1							1	1			1					1		1
3		1	1				1		1				1	1		1		1				
4					1			1											1			
5										1											1	
6																	3					
7						2																
8																						

(A)

Support	0.6												0.7											
Graph Size	1	2	3	4	5	6	7	8	9	10	11	12	1	2	3	4	5	6	7	8	9	10	11	12
2	1							1		1			1							1		1		
3			2	1	1						1				2	1	1							
4									1												1			2
5		2									3	4		1									1	
6											3												1	
7																		6						
8																		4						
9						2																		
10						8													10					
11							10																	

(B)

Figure 1: The tables above show the number of times that a pattern of a given group, support and size appears. Table (A) represents patterns from the serine protease dataset, where the largest patterns at supports 0.6 and 0.7 are highlighted. Table (B) represents patterns from the BCL-2 dataset. The largest patterns from correspondent groups are highlighted with the same color.

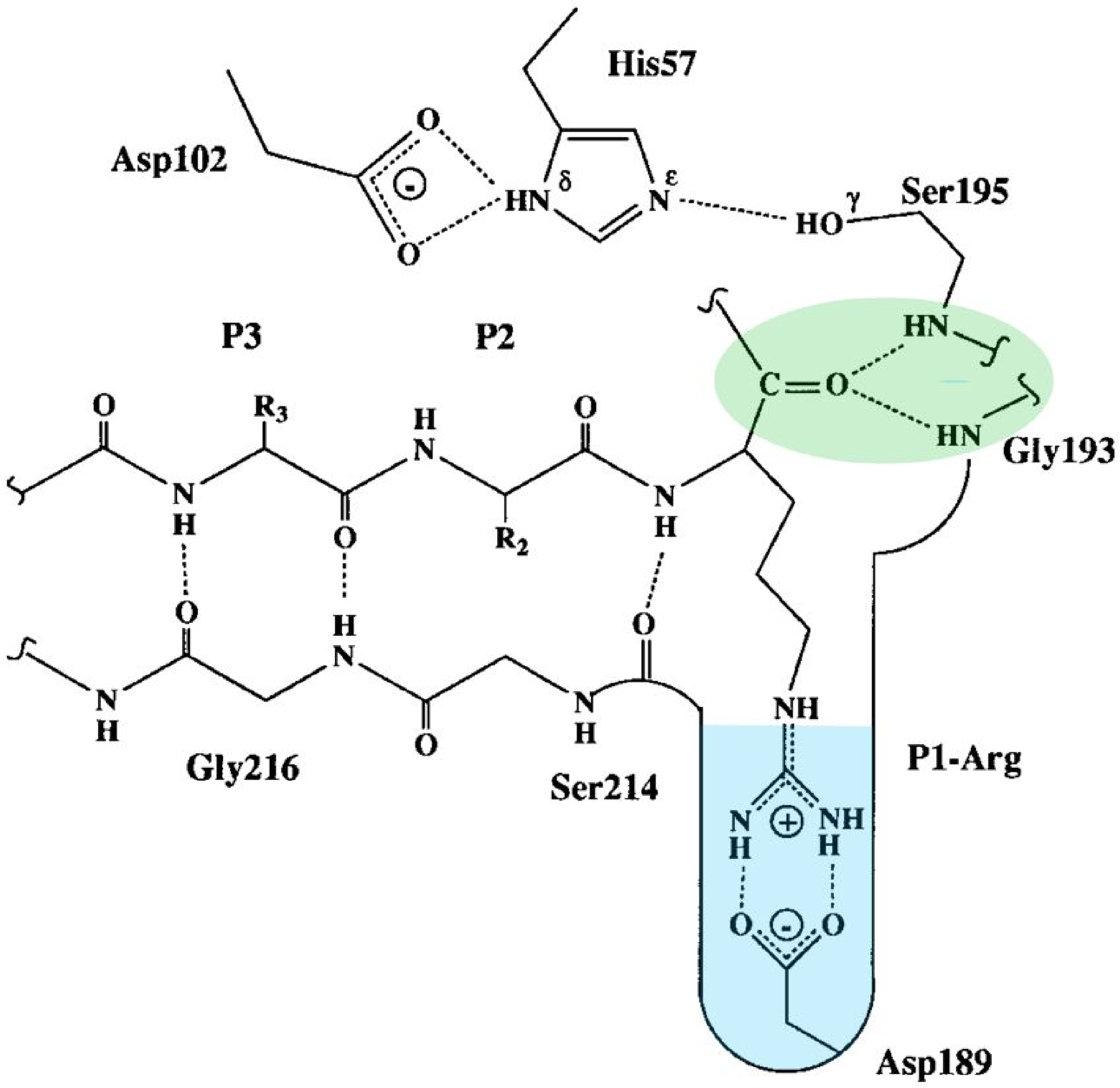

Figure 2: Chemical mechanism of catalysis for trypsin. The S1 pocket is highlighted in blue, with Asp189 at the base interacting with an arginine residue at position P1. Residues from the catalytic triad are shown as Ser195, His57 and Asp102. The oxyanion hole is highlighted in green, formed by hydrogen bond interactions from the carbonyl group of Arg-P1 with atoms from Ser195 and Gly193. Adapted from “Evolutionary divergence of substrate specificity within the chymotrypsin-like serine protease fold” by J. J. Perona and C. S. Craik, 1997, *Journal of Biological Chemistry*, 272.48, p. 29987-29990
